# Long-term live cell cycle imaging of single *Cyanidioschyzon merolae* cells

**DOI:** 10.1007/s00709-020-01592-z

**Published:** 2021-01-05

**Authors:** Takako M. Ichinose, Atsuko H. Iwane

**Affiliations:** 1 Center for Biosystems Dynamics Research, Laboratory for Cell Field Structure, Riken, 3-10-23, Kagamiyama, Higashihiroshima, 739-0046 Japan; 2 Graduate School of Frontier BioScience for Systems Science of Biological Dynamics, Osaka University, 1-3, Suita, 565-0871 Japan; 3 Graduate School of Integrated Sciences for Life, Hiroshima University, 3-10-23, Kagamiyama, Higashihiroshima, 739-0046 Japan

**Keywords:** Long-term time-lapse observation, Live cell cycle imaging, *Cyanidioschyzon merolae*, β-Tubulin

## Abstract

Live cell imaging by fluorescence microscopy is a useful tool for elucidating the localization and function of proteins and organelles in single cells. Especially, time-lapse analysis observing the same field sequentially can be used to observe cells of many organisms and analyze the dynamics of intracellular molecules. By single-cell analysis, it is possible to elucidate the characteristics and fluctuations of individual cells, which cannot be elucidated from the data obtained by averaging the characteristics of an ensemble of cells. The primitive red alga *Cyanidioschyzon merolae* has a very simple structure and is considered a useful model organism for studying the mechanism of organelle division, since the division is performed synchronously with the cell cycle. However, *C. merolae* does not have a rigid cell wall, and environmental changes such as low temperature or high pH cause morphological change and disruption easily. Therefore, morphological studies of *C. merolae* typically use fixed cells. In this study, we constructed a long-term time-lapse observation system to analyze the dynamics of proteins in living *C. merolae* cells. From the results, we elucidate the cell division process of single living cells, including the function of intracellular components.

**Supplementary Information:**

The online version contains supplementary material available at 10.1007/s00709-020-01592-z.

## Introduction

Live cell imaging is a useful technique for studying the localization and dynamics of molecules in living cells. Especially, time-lapse imaging enables tracking of the temporal and spatial dynamics of molecules during cell cycle progression and the corresponding signal response pathways to elucidate their function. *Cyanidioschyzon merolae* (*C. merolae*) is a primitive eukaryotic unicellular red alga isolated from a sulfate hot spring near Naples and believed to retain the properties obtained immediately after mitochondria and plastids were born by symbiosis. It is an autotroph that can reduce carbon dioxide to make organic compounds for biosynthesis and the storage of chemical energy. Its cell has a very simple structure, with only one nucleus, mitochondrion, plastid, and peroxisome. The division of each of these organelles is synchronized to the progression of the cell cycle, ensuring that the organelles are distributed equally to the two daughter cells (Kuroiwa et al. 1998; Misumi et al. 2005). Moreover, the cell cycle of *C. merolae* can be synchronized with the cycle of light (Suzuki et al. 1994). Thus, *C. merolae* is considered a useful model organism to study organelle division.

The genome sequences of *C. merolae* in the nucleus, plastid, and mitochondrion have already been determined (Ohta et al. 1998, 2003; Matsuzaki et al. 2004; Nozaki et al. 2007). Furthermore, many biological functional analysis techniques have been applied, such as gene transfer to insert target genes into the genome using homologous recombination, to study the organism (Minoda et al. 2004; Fujiwara et al. 2013). By these techniques and two uracil-auxotrophic strains (M4 and T1), strains stably expressing a desired molecule can be efficiently constructed, allowing for detailed functional analysis (Minoda et al. 2004; Taki et al. 2015).

*C. merolae* does not have a rigid cell wall, and thus environmental changes such as low temperature or high pH cause its morphological change or destruction easily. Therefore, morphological studies use cells chemically fixed with glutaraldehyde. To analyze the temporal and spatial functions of target molecules in single cells, however, live cell imaging technology is desired. Recently, Sumiya et al. (2016) succeeded in constructing a time-lapse observation system, in which the cells are sandwiched between two coverslips, to observe individual cells for 24 h continuously. In order to further understand the temporal structural changes and molecular movements during organelle division and the cell cycle, it is necessary to observe the cells continuously from immediately before one cell division to the end of the next cell division. That is, continuous observation of more than twice the doubling time is desired.

In this study, we established a system that allows for the long time-lapse analysis and live cell imaging of *C. merolae*. *C. merolae* cells inhabit the rocky areas of hot springs and in the laboratory are usually cultured under liquid shaking conditions to supply carbon dioxide. However, we confirmed *C. merolae* grown on flat plates in static culture adhered to the substrate and could undergo cell division similarly as in liquid suspension culture. Furthermore, we established a *C. merolae* cell strain that stably expresses β-tubulin proteins, one of the main components of microtubules, fused to *superfolder* GFP (*sf*GFP), a GFP with high thermal stability, for live cell time-lapse imaging (Pédelacq et al. 2006). Live cell imaging by fluorescence microscopy revealed β-tubulin localization depended on the cell cycle. Our live cell long-term time-lapse imaging system allows further function analysis of molecules in *C. merolae*.

## Materials and methods

### Cell cultures

The wild-type *C. merolae* strain, MO, and the uracil-auxotrophic mutant, T1, were used in this study. In T1, the *URA5.3* gene is completely deleted, which allows a backgroundless selection of transformants (Taki et al. 2015). The strains were cultured in gyratory culture (120 rpm) at 42 °C under continuous light (50 μmol/m^2^/s) in MA2 medium (pH 2.5) and MA2 medium containing uracil (0.5 mg/ml), respectively (Ohnuma et al. 2008).

### Construction of plasmids and recombinant cells

The β-tubulin (CMN263C) gene and *sf*GFP gene were amplified by PCR from *C. merolae* 10D total DNA and the pNRp:: *sf*GFP plasmid (Fujiwara et al. 2015) using the primer sets tgtgtttcttcgttcgttgaccatgcgtgagatactgcatattcaggttg and GTTTGTACTGTGGTAACAGG TCcatgatcgagctttcgatagctgttc, and ctgtgtttcttcgttcgttgaccATGAGCAAGGGCGAGGAG CTGTTCACC and TTACTTGTACAGCTCGTCCATG, respectively. The pD184-O250-EGFP-Ura_cm-cm_ vector (Fujiwara et al. 2013) was amplified by PCR with the primer set GGACGAGCTGTACAAGTAAactagctatttatctggtaca and ggtcaacgaacgaagaaacacagag to replace the EGFP gene with the *sf*GFP gene and then constructed with the amplified *sf*GFP gene using Gibson Assembly Master Mix (NEB). The resultant pD184-O250-*sf*GFP-Ura_cm-cm_ vector was amplified by PCR with the primer set GACCTGTTACCACAGTACAAAC and ggtcaacgaacgaagaaacacagag. The β-tubulin gene was inserted at the 5′ end of the *sf*GFP gene of the pD184-O250-*sf*GFP-Ura_cm-cm_ vector. The pD184-O250-β-tubulin-*sf*GFP-Ura_cm-cm_ plasmid was used as a template to amplify DNA fragments with the primer set CACCATCACCATCACGCGTGAGTCAG TTCACTGAC and AAGCTCAGCTAATTACAGCTTGCTGACCTTACCC for the transformation.

To establish a stable transformant, 2 μg of the PCR fragments amplified by the high-fidelity PCR polymerase PrimeSTAR Max (Takara) was introduced into *C. merolae* T1 using PEG-mediated protocols (Ohnuma et al. 2008; Imamura et al. 2010). The cells were mixed with PCR fragments and PEG and then cultured overnight as described above with 5% CO_2_. The next day, the cells were washed with MA2 medium and suspended, then spotted on starch spots on a MA2 plate containing no uracil. The transformed cells were incubated until colonies formed under continuous white light at 40 °C. Colonies were picked up and suspended in MA2 medium. The insertion of β-tubulin-*sf*GFP into the *C. merolae* T1 genome was confirmed by genomic PCR using PCR polymerase KOD Fx Neo (TOYOBO) with the primer set CACCATCACCATCACGCGTGAGTCAGTTCACTGAC and AAGCTCAGCTAATTACAGCTTGCTGACCTTACCC at the insertion region.

### Time-lapse analysis

Time-lapse analysis was carried out using a BIOREVO BZ-9000 (KEYENCE) fluorescence microscope. To control the temperature (42 °C) and CO_2_ concentration (5%), we used a stage top incubator (Tokaihit). Because *C. merolae* requires light for its growth, we set a white LED light on the stage top incubator. The light intensity was set at 50 μmol/m^2^/s. Cells were diluted to an OD_750_ of 0.03, seeded on an μ-Dish^35 mm, high^ (ibidi), and put in the stage top incubator. Time-lapse observation was started 4 h after seeding the cells in order to allow the cells to adhere to the bottom of the ibidi dish. Time-lapse images were obtained every 30 min for 4 days. In order to minimize defocusing during the long time-lapse observation, 60 to 80 Z-stack images were taken at each time point. The most focused images were selected using the analysis application BZ-II Analyzer (KEYENCE), and time-lapse continuous images were acquired. In order to perform two consecutive time-lapse analyses, cells that reached the log phase in the first time-lapse analysis were resuspended, diluted, and then seeded in a new ibidi dish. The second time-lapse analysis was started 2 h after we confirmed that the cells adhered to the bottom of the ibidi dish.

### Doubling time measurements

The obtained continuous images were loaded to an image analysis application, ImageJ (US National Institutes of Health, Bethesda, MD, USA), and the number of frames was counted until individual cells underwent cytokinesis. The doubling time was calculated by multiplying the number of frames by the measurement interval of 30 min.

### Live cell time-lapse imaging

Live cell time-lapse images were captured using a confocal fluorescence microscope (OLYMPUS FV3000). As the objective lens, we used USLSAPO 100XS (OLYMPUS) with silicon oil, because regular oil ruptures the bottom membrane of the ibidi dish. β-tubulin-*sf*GFP T1 cells were seeded on the ibidi dish and incubated at 42 °C, 5% CO_2_. We set a white LED light on the stage top incubator. The light intensity was set at 15 μmol/m^2^/s. Time-lapse imaging was started 4 h after the cell seeding. The time-lapse images were obtained every 15 min for 40 h.

## Results

### Construction of time-lapse observation system for *C. merolae*

**Fig. 1:**
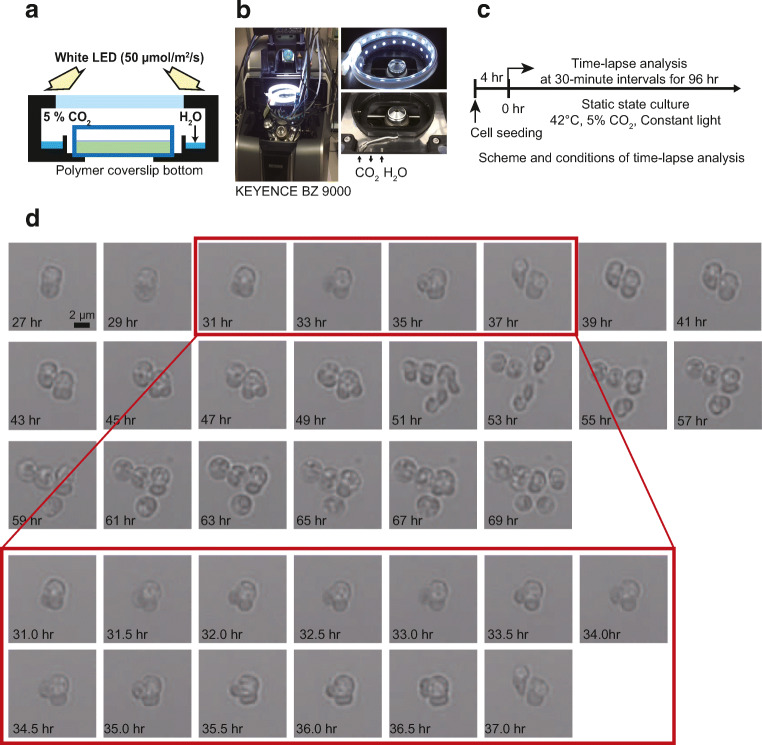
Culture conditions of *C. merolae* and construction of the chamber for time-lapse imaging. (a, b) To perform a functional analysis of individual cells, static culture conditions and a chamber for time-lapse imaging were constructed. The inside of the thermostatic chamber is kept at 42 °C and 5% CO_2_. *C. merolae* also needs light to carry out photosynthesis, so white LED light is illuminated into the thermostatic chamber. (c) The scheme and conditions of the time-lapse imaging. (d) Time-lapse images were obtained every 30 min. The number in the lower left indicates the elapsed time from when the time-lapse imaging began. In the red frames, morphological changes every 30 min from stage II (S phase) to stage V (M phase) are shown

In order to analyze the dynamic localization of molecules involved in the cell cycle in individual cells, a time-lapse observation system was constructed (Fig. 1a, b). The chamber was installed inside a fluorescence microscope to maintain the temperature and CO_2_ concentration. *C. merolae* also needs light for growth, so an on/off controllable white LED light was introduced on top of the chamber. *C. merolae* is usually cultured by liquid shaking culture; however, in order to observe individual cells sequentially, a vessel for static culture was applied. To observe living cells, an objective lens with high magnification and high numerical aperture and a glass bottom dish are typically used, since the thickness and refractive index of the dish are equivalent to those of the cover glass. This was not possible with *C. merolae* cells, because the cell adhesion was too weak to observe the same cells for a long time. Therefore, we needed to try several types of polymer coverslip bottom dishes and pre-coated glass bottom dishes*.* These polymer coverslip bottom dishes and glass bottom dishes are widely used for imaging weakly adhesive cells such as primary cultured cells. Of those tested, the most useful was the ibidi dish (see “Materials and methods”) as shown in supplement Table 1 (Online Resource 1). The plastic film used for the bottom surface has a thickness of 175 μm and a refractive index of 1.5, which are similar properties to the cover glass. The ibidi dish also has gas permeability, which enables the exchange of carbon dioxide and oxygen during the cell culture. *C. merolae* cells were inoculated on the plastic film for 4 h and confirmed to adhere to the bottom stably. The cells were cultured for 4 days in a chamber maintained at 42 °C, 5% CO_2_, and constant 50 μmol/m^2^/s light, and bright field time-lapse images were obtained every 30 min (Fig. 1c). From the obtained continuous images, 100 cells were selected, and the morphological changes were observed. Most cells were able to perform cell division three times on the dish. Figure 1d shows that cells that completed the first cell division typically underwent two more cell divisions. This observation indicated that *C. merolae* was able to grow and perform cell division while attached to the bottom of an ibidi dish.

### Cell division on ibidi dishes

**Fig. 2:**
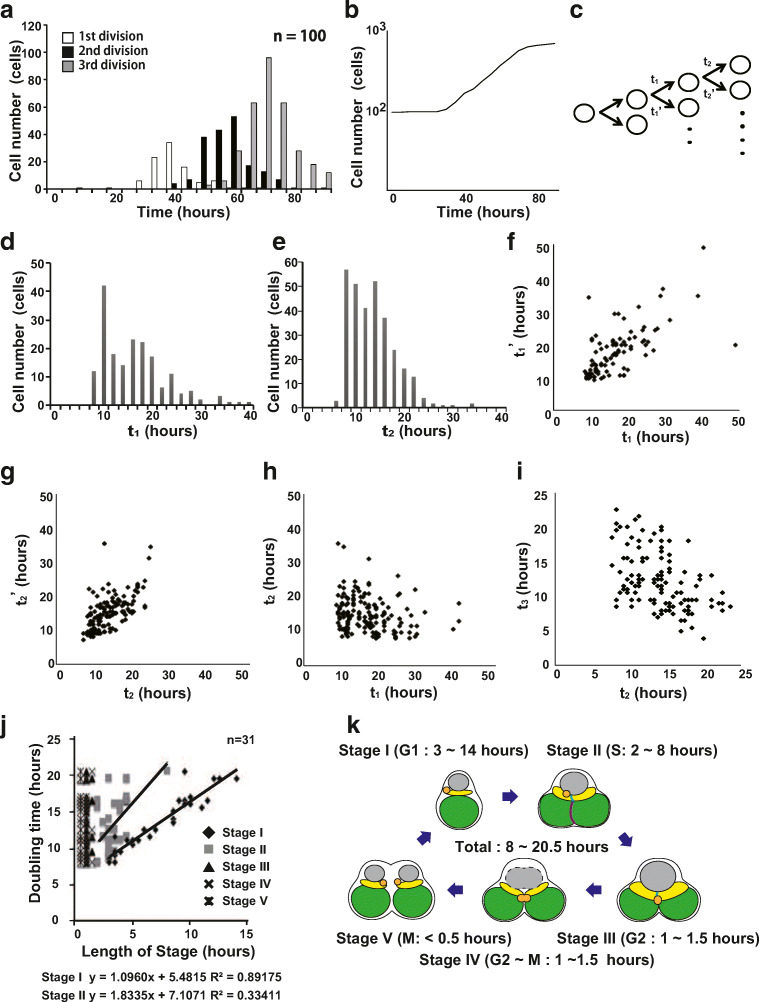
Measurement of generation time. (a) Cell division time from the start of the time-lapse imaging. (b) The growth curve obtained from the number of cells per observation field. The doubling time of cells was calculated from the period in which linear cell growth (about 16.9 h). (c–e) The distribution of the generation time. (d) The duration from the first division to the second division (*t*_1_). (e) The duration from the second division to the third division (*t*_2_). (f, g) A correlation analysis between the generation time of two daughter cells. (f) *t*_1_ and *t*_1_’. (g) *t*_2_ and *t*_2_’. Both *t*_1_ and *t*_1_’, and *t*_2_ and *t*_2_’ showed positive correlations. (h, i)A correlation analysis between the generation time of the parent cell and the generation time of the daughter cell. (h) *t*_1_ and *t*_2_. (i) *t*_2_ and *t*_3_. The generation time of the parent cell and the generation time of the daughter cell showed a weak inverse correlation. (j) A correlation analysis between the duration of each stage and the generation time indicate that the generation time depended on the length of stage I. (k) The cell cycle was classified into five stages from the morphology of the cells (Suzuki et al. 1994), and the duration of each stage was measured. The duration of each stage in static culture is shown in parentheses

To investigate whether adhesion to the dish inhibited cell division, the cell doubling time was analyzed. Most of the cells seeded on the dish did not start dividing for about 40 h, but once division started, second and third divisions were regularly observed (Fig. 2a). Figure 2b shows the growth curve based on the number of cells per observation field. The doubling time of the cells calculated from the period in which a linear cell growth was observed was about 16.9 h. This duration is shorter than the previously reported cell cycle duration of 19 h (Fujiwara et al. 2009; Imoto et al. 2010). Analyzing the distribution of the duration from the first division to the second division (*t*_1_) and the duration from the second division to the third division (*t*_2_), we found *t*_1_ and *t*_2_ were distributed between 8 and 24 h (Fig. 2c–e). This indicates that there is a gap between the generation times of individual cells. The duration until the two daughter cells underwent the next division (*t*_1_ and *t*_1_’, *t*_2_ and *t*_2_’) was positively correlated, indicating that the two daughter cells have the same properties regarding cell division (Fig. 2f, g).

The relationship between the generation time of the parent cell and the generation time of the daughter cell was also analyzed, revealing *t*_1_ and *t*_2_ have a weak inverse correlation (the correlation coefficient; − 0.208). We further showed that the generation time of daughter cells that divide from a parent cell with a short generation time is longer than that of the parent cell, and conversely the generation time of daughter cells that divide from a parent cell with a shorter generation time tends to be shorter (Fig. 2h). This weak inverse correlation tends to be more pronounced when the parent cell generation time is long. The correlation coefficient was 0.008 when the parent cell generation time was shorter than 14 h, whereas it was − 0.199 when the generation time was 14 h or more. This inverse correlation was also observed between the duration of the second division and the third division (*t*_2_) and the duration of the third division and the fourth division (*t*_3_) (the correlation coefficient; − 0.470) (Fig. 2i). The cell cycle has been classified into five stages based on the cell morphology (Suzuki et al. 1994). The durations of stages I, II, III, IV, and V were 3~14 h, 2~8 h, 1~1.5 h, 1~1.5 h, and < 0.5 h, respectively (Fig. 2j, k). A correlation analysis between the duration of each stage and the generation time indicated that the generation time depended on the length of stage I.

**Fig. 3:**
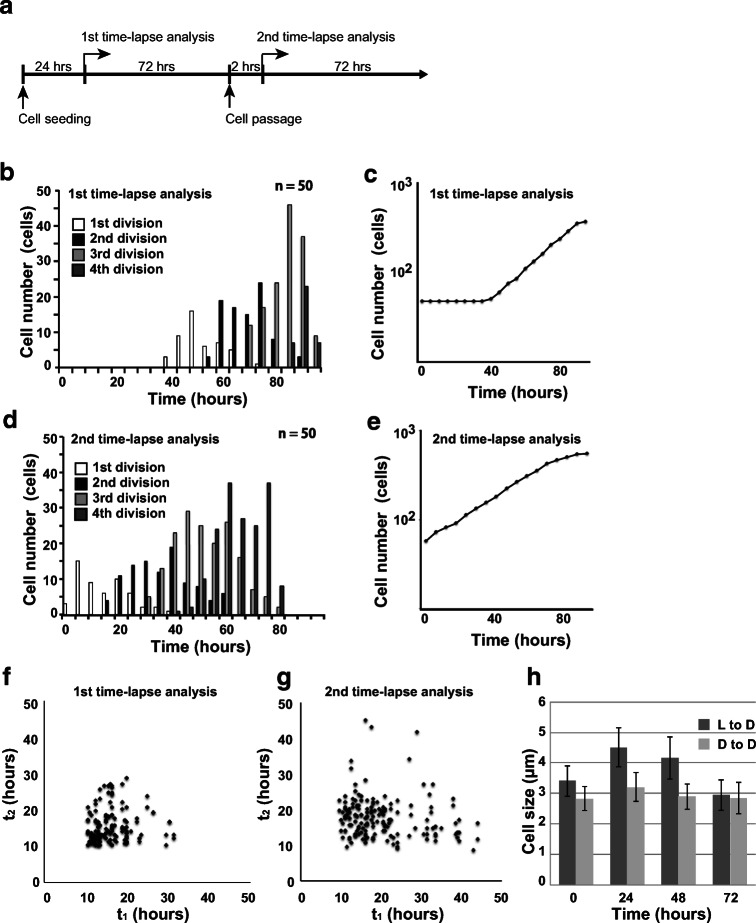
Measurement of the generation time of passaged cells. (a) The scheme of two consecutive time-lapse analyses. (b) Cell division time from the start of the first time-lapse imaging. (c) The growth curve measured from the number of cells per observation field of the first time-lapse analysis. The doubling time of cells was calculated from the period in which a linear cell growth was observed (about 17.8 h). (d) Cell division time from the start of the second time-lapse imaging. The cells immediately started to proliferate without arresting the cell cycle. (e) The growth curve measured from the number of cells per observation field of the second time-lapse analysis. The doubling time of cells was calculated from the growth curve (about 17.3 h). (f) A correlation analysis between the generation time of the parent cell and the generation time of the daughter cell in the first time-lapse analysis. (g) A correlation analysis between the generation time of the parent cell and the generation time of the daughter cell in the second time-lapse analysis. The generation times of the parent cell and daughter cell showed a weak inverse correlation. (h) The diameters of the cells seeded on a dish from the liquid culture (L to D) and the cells passaged from a dish to a dish (D to D). The cell diameter increased in L to D, but hardly changed in D to D

In order to investigate whether the cells once acclimated to the environment on the dish retain their properties, the cells that reached the log phase in the first time-lapse analysis were passaged, and the second time-lapse analysis was performed (Fig. 3a–g). The cells passaged from the dish of the first time-lapse analysis immediately started to proliferate without arresting the cell cycle (Fig. 3d, e). The doubling time of the cells calculated from the growth curve was about 17.3 h. This value is almost the same as the doubling time in the log phase of the first time-lapse analysis. In the first time-lapse analysis, no inverse correlation between the generation time of parent cells and daughter cells was observed as a whole (the correlation coefficient; 0.108), but an inverse correlation was observed when the generation time of parent cells was 14 h or more (the correlation coefficient; − 0.144) (Fig. 3f). In the second time-lapse analysis, the generation times of the parent cell and daughter cell showed a weak inverse correlation (the correlation coefficient; − 0.186). Just as the first time-lapse analysis, this tendency was stronger for longer generation times of the parent cells (less than 14 h; 0.0861, 14 h or more; − 0.203) (Fig. 3g). We therefore inferred that the generation time of *C. merolae* does not converge to the average doubling time obtained from the growth curve and repeatedly fluctuates in each generation.

As shown in the first time-lapse analysis, cells immediately after being seeded on a dish stopped their cell cycle. The size of the cells increased during this period. The diameters of the cells seeded on a dish from the liquid culture (L to D) and the cells passaged from a dish to a dish (D to D) were compared every time point (0, 24, 48, and 72 h) (Fig. 3h). The diameter of the L to D cells increased after 24 h, but then decreased with cell division. On the other hand, D to D cells divided immediately after seeding, and their diameter remained almost unchanged thereafter. This data suggest that the cells take time to respond to environmental change, but the cells once acclimated to the dish inherit the properties of the daughter cells. These results suggest that the cell division of *C. merolae* is not inhibited by adhesion to the ibidi dish and that our imaging system improves the measurement accuracy of the generation time of individual cells.

### Live cell time-lapse imaging with fluorescence microscopy

**Fig. 4:**
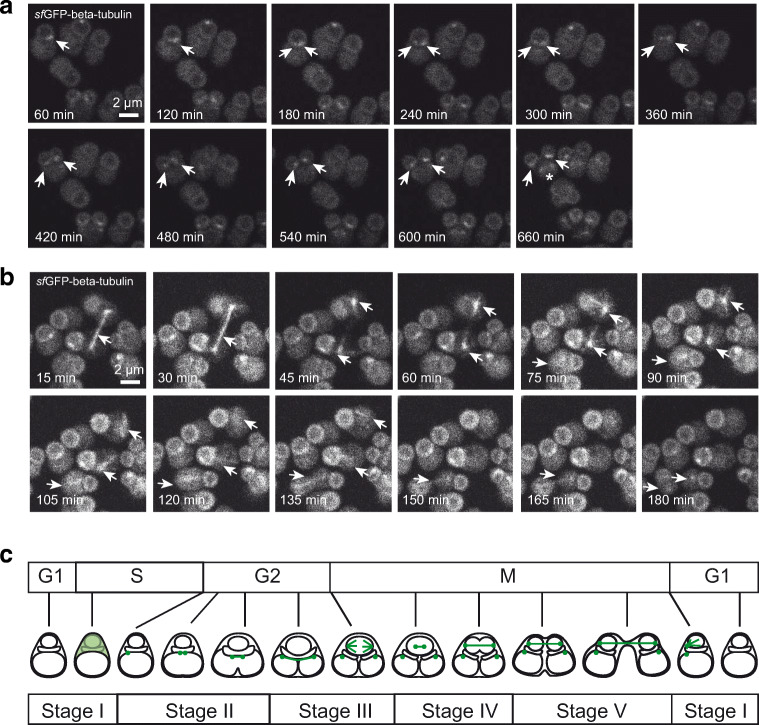
Live cell imaging of cell strains stably expressing β-tubulin-*sf*GFP. Time-lapse images were obtained every 15 min. The cells were cultured for 40 h in a chamber maintained at 42 °C, 5% CO_2_, and constant 15 μmol/m^2^/s light. The number in the lower left indicates the elapsed time from when the time-lapse imaging was started. (a) β-tubulin-*sf*GFP was localized at one edge of a mitochondrion in G2 phase (images with one arrow). Then, β-tubulin-*sf*GFP was divided into two prior to mitochondrion division (images with two arrows). In M phase, another β-tubulin-*sf*GFP focus appeared near the nucleus (white asterisk). (b) The spindle was stretched to both poles linearly during mitosis (white arrows). The fluorescence was detected until after cytokinesis, but disappeared at the time of G1 phase. (c) A model for microtubule localization and morphology changes during the cell cycle. Microtubules are shown in green

To investigate the dynamic localization of a molecule in an individual cell, a recombinant *C. merolae* strain expressing GFP stably was constructed. Since *C. merolae* is cultured at 42 °C, *superfolder* GFP (*sf*GFP), which has a higher thermal stability than EGFP, was used as the fluorescent protein tag. In many eukaryotic cells, microtubules are a major cytoskeleton component. They are cylindrical fibers consisting of α-tubulin and β-tubulin heterodimers. Microtubules repeat polymerization and depolymerization and play important roles in maintaining cell shape, cell division, and intracellular transport. In mitosis, microtubules and microtubule-binding proteins play a central role in chromosomal arrangement and separation. In *C. merolae*, microtubules are involved in chromosome segregation in the nucleus, mitochondrial morphology, and the segregation and division of single membrane-bound microbodies. Since *C. merolae* tubulin is expressed only during a certain period of the cell cycle and is organized only during cell division as microtubules, it is presumed that microtubules do not maintain *C. merolae* cell morphology (Nishida et al. 2009; Imoto et al. 2010). A stable strain expressing β-tubulin (CMN263C)-*sf*GFP (β-tubulin-*sf*GFP T1) was prepared by inserting β-tubulin-*sf*GFP gene constructed in pD184-O250-*sf*GFP-Ura_cm-cm_ vector using homologous recombination into the D184-D185 locus of the genome. β-tubulin-*sf*GFP is expressed from the APCC(CMO250) promoter. β-tubulin-*sf*GFP T1 cells were cultured on an ibidi dish at 42 °C, 5% CO_2_, and the localization of the molecules was observed using confocal laser microscopy as shown in the Supplement movie 1 (Online Resource 2). Time-lapse images were obtained every 15 min for 40 h. In the G2 phase, β-tubulin-*sf*GFP was localized at one edge of the mitochondrion, then divided into two locations prior to mitochondrion division (Fig. 4a). In the M phase, the two β-tubulin-*sf*GFP foci appeared near the nucleus. Next, a spindle was formed and stretched to both poles linearly. The formed spindle extended between the two daughter cells and eventually split. Fluorescence was observed in the daughter cells until 90 min after cytokinesis and then disappeared during the G1 phase (Fig. 4b). This observation is consistent with a previous report that showed there are two types of bipolar microtubules that form at the mitochondrial and nuclear edges in the cell cycle (Imoto et al. 2010). In Fig. 4c, a model for microtubule localization and morphology changes during the cell cycle obtained from these observations is shown.

## Discussion

### Cytoplasmic division in static culture

*C. merolae* provides an attractive early evolutionary model for studying the functions of various molecules. However, most experimental systems use fixed cells, preventing observation of the dynamics. Here, we report a new imaging system that allows us to observe the dynamics of target molecules in single living cells. As a proof of concept, we applied our system to β-tubulin, showing how microtubule localization correlates with different stages of the cell cycle. One technical modification necessary for this observation was the choice of the glass dish (Supplement Table 1). Because long-term observation can affect the adhesion of the cells to the dish bottom, we tested various polymer-coated dishes, finding that the ibidi μ-Dish^35 mm, high^ was best. Most cells can divide on the ibidi dish. Even then, some cells, particularly hypertrophied cells, failed to undergo cytokinesis, possibly because the movement was restricted by adhesion to the bottom surface of the dish. On the other hand, cells capable of cell division began to twist and/or flutter significantly at the individual daughter’s cell domain which daughter cells divide with expansion and contraction of microtubules before cytokinesis (Online Resource 2). Although details are unknown, these observations suggest that not only the increase or decrease and localization of proteins are directly involved in cytokinesis but also the unknown dynamic motility of cells may be involved in cytokinesis.

On the dish, the doubling time of cells calculated from the growth curve was about 16.9 h. This duration is shorter than the previously reported cell cycle duration of 19 h (Fujiwara et al. 2009; Imoto et al. 2010). This reduction might be because, in our flat culture, each cell is cultured in a monolayer state so that the cell can always utilize light energy without being shaded by other cells, which is different from the suspension culture system. However, most cells took 40 to 50 h until beginning the first division upon seeding on the dish. The only exceptions were those not at stage I when seeded, possibly because *C. merolae* cells at stage I might need time to adapt to the environmental change of the culture before starting cell proliferation. The cause of the stage I exception requires further analysis of several cell cycle checkpoint-related factors. The diameter of *C. merolae* cells cultured under liquid shaking conditions is about 3 μm, but after seeding in the dish in our experiments, the cells grew to approximately 5 μm without cell division. The diameter then returned to 3 μm during the two rounds of cell division. When the cells that repeatedly divided on the dish were resuspended and replated again on the dish, they continued to proliferate without stopping the cell division or expansion. These observations suggest that the cells once acclimated to the dish inherit the properties of the daughter cells.

To study the cell cycle of *C. merolae*, the cells need to be synchronized with a 12-h light/12-h dark cycle (Suzuki et al. 1994). We originally designed the imaging device to include this synchronization feature. However, the seeded cells stopped dividing for 40–50 h and then started dividing at the same time. Therefore, the cell cycle was synchronized despite the continuous light conditions during the observation period.

The averaged doubling time obtained from the growth curve was approximately 16.9 h, while the generation time of individual cells was approximately 8 to 24 h. A positive correlation was found in the generation times of daughter cells, but a weak inverse correlation was found in the generation times of parent cells and daughter cells. This inverse correlation did not change even as the generation progressed, and fluctuations in the generation time were sustained. This data indicates that the generation time of *C. merolae* does not converge to the average doubling time obtained from the growth curve and repeatedly fluctuates in each generation. These results suggest that our imaging system can characterize individual cells rather than averaged properties.

### Live cell time-lapse imaging using fluorescence microscopy

In previous reports, the intracellular localization of various molecules in *C. merolae* was mainly identified by using immunofluorescent staining (Nishida et al. 2004). Immunofluorescent staining is excellent for the simultaneous observation of several intracellular molecules and the analysis of interactions between molecules. However, since the immunofluoresecent staining requires a chemical fixation step, it is difficult to prevent protein denaturation and the loss of soluble proteins. On the other hand, live cell imaging enables an analysis of the protein function and localization changes within individual cells. By observing single cells of a *C. merolae* recombinant strain stably expressing β-tubulin-sfGFP, we found that β-tubulin localization corresponded to cell cycle progression. Furthermore, using this system, we could observe the same cells and their daughter cells for a long time (~ 4 days), indicating the usefulness of our system for observing the dynamics and localization of substances inherited from parent cells. Previously, the modification of multiple genes on the genome was difficult due to limited selection markers. In recent years, the development of new markers (Fujiwara et al. 2017; Zienkiewicz et al. 2017a; Zienkiewicz et al. 2017b) and a marker recycling system (Takemura et al. 2018) has made it possible to target two or more genes. Adding these innovations to our fluorescence imaging system will enable the observation of multiple proteins simultaneously.

In immunostaining experiments using fixed cells, various fluorescent dyes and fluorescent proteins have been used to identify target molecules. However, in long time-lapse imaging, we found it necessary to avoid using wavelengths that excite the autofluorescence of plastids, because the viability of the cells was lost as the autofluorescence of the plastids faded (data not shown). Although the reason is not clear, long-time irradiation of the excitation light may compromise the function and structure of the plastids. Finally, for our time-lapse observation system, we used the ibidi μ-Dish^35 mm, high^, but this dish adds some constraints compared to other dishes. The first is that normal lens oil damages the membrane at the bottom of the dish. For this reason, silicone oil and silicone oil lenses have to be used. The second is the focus shift due to the elasticity of the membrane. Although this shift can be alleviated to some extent by the microscope’s focus-following system, sometimes the focus deviates beyond the limits of the system. Therefore, it is necessary to set the Z-stack widely than when using a conventional glass-bottom dish. To conclude, we proposed a long-term time-lapse imaging system for *C. merolae*. Since this system can control temperature, carbon dioxide concentration, and light intensity, it should be applicable to functional studies of other intracellular molecules in many single-cell algae.

## Supplementary information

Supplement Table 1 Comparison of several dishes and their coating materials. Several types of commercial polymer coverslip bottom dishes and pre-coated glass bottom dishes were compared. Cell adhesion was weak in all glass bottom dishes, making them unsuitable for long-time time-lapse analysis. On the other hand, polymer coverslip bottom dish B was too adhesive and inhibited cell division. Polymer coverslip bottom dish A (ibidi) was best for our long-time time-lapse analysis. (PDF 47 kb)

Supplement movie 1 *C. merolae* mitosis (Time-Lapse). Green, β-tubulin; red, auto-fluorescence of chlorophyll. This video replays a 30-h observation of *sf*GFP-β-tubulin-expressing T1 recombinant cells undergoing mitosis at 4500 times faster than real time. (AVI 3376 kb)

## Data Availability

Yes
