# Supplementary material for: Long-term live cell cycle imaging of single Cyanidioschyzon merolae cells
Source: Protoplasma. 2021 Jan 5;258(3):651–60. doi: 10.1007/s00709-020-01592-z (PMC8052221; doi:10.1007/s00709-020-01592-z)
Supplement: Supplementary file 1 — Comparison of several dishes and their coating materials. Several types of commercial polymer coverslip bottom dishes and pre-coated glass bottom dishes were compared. Cell adhesion was weak in all glass bottom dishes, making them unsuitable for long-time time-lapse analysis. On the other hand, polymer coverslip bottom dish B was too adhesive and inhibited cell division. Polymer coverslip bottom dish A (ibidi) was best for our long-time time-lapse analysis. (PDF 47 kb) [file 709_2020_1592_MOESM1_ESM.pdf]

Dish and coating	Cell Adhesiveness	Cell morphology	Cell dispersibility	High resolution analysis
**Plastic culture dish**	+ + +	+ + +	+ + +	+
**Polymer coverslip bottom dish A (ibidi)**	+ + +	+ + +	+ + +	+ + +
**Polymer coverslip bottom dish B**	+ + +	+	+ +	+ + +
**Glass bottom dish**	+	+ + +	+	+ + +
**+ Lysin coat**	+	+ + +	+	+ + +
**+ Laminin coat**	+	+ + +	+	+ + +
**+ Gellan gum coat**	+	+ + +	+	+ + +

+ + +; Good, + +; Average, +; Poor
